# Results from a randomised controlled pilot study of the Better Conversations with Primary Progressive Aphasia (BCPPA) communication partner training program for people with PPA and their communication partners

**DOI:** 10.1186/s40814-023-01301-6

**Published:** 2023-05-23

**Authors:** A Volkmer, H Walton, K Swinburn, A Spector, J. D Warren, S Beeke

**Affiliations:** 1 Division of Psychology and Language Sciences, University College London, London, UK; 2 Department of Applied Health Research, UCL, London, UK; 3 Dementia Research Centre, Department of Neurodegenerative Disease, UCL Institute of Neurology, University College London, London, UK

**Keywords:** Dementia, Primary progressive aphasia, Communication partner training, Communication, Rehabilitation of speech and language disorders

## Abstract

**Background:**

There has been a growing focus on functional communication interventions for primary progressive aphasia (PPA). These interventions aim to support individuals to participate in life situations. One such intervention, communication partner training (CPT) aims to change conversation behaviours in both the person with PPA and their communication partner (CP). CPT has a growing evidence base in stroke aphasia; however, these programmes are not designed to meet the needs of people with progressive communication difficulties. To address this, the authors developed a CPT program entitled Better Conversations with PPA (BCPPA) and undertook a pilot trial to establish for a future full trial; predicted recruitment rates, acceptability, an assessment of treatment fidelity and an appropriate primary outcome measure.

**Methodology:**

This was a single-blind, randomised controlled pilot study comparing BCPPA to no treatment, delivered across 11 National Health Service Trusts in the UK. A random sample of eight recordings of local collaborators delivering the intervention were analysed to examine fidelity. Participants completed feedback forms reporting on acceptability. Pre- and post-intervention measures targeted conversation behaviours, communication goals and quality of life.

**Results:**

Eighteen people with PPA and their CPs (9 randomised to BCPPA, 9 randomised to no treatment) completed the study. Participants in the intervention group rated BCPPA positively. Treatment fidelity was 87.2%. Twenty-nine of 30 intervention goals were achieved or over-achieved and 16 of 30 coded conversation behaviours demonstrated change in the intended direction. The Aphasia Impact Questionnaire was identified as the preferred outcome measure.

**Conclusion:**

The first randomised controlled UK pilot study of a CPT program for people with PPA and their families demonstrates BCPPA is a promising intervention. The intervention was acceptable, treatment fidelity high and an appropriate measure identified. Results of this study indicate a future RCT of BCPPA is feasible.

**Trial registration:**

Registered 28/02/2018 ISRCTN10148247.

**Supplementary Information:**

The online version contains supplementary material available at 10.1186/s40814-023-01301-6.

## Key messages regarding feasibility

- What uncertainties existed regarding the feasibility?

This study addressed uncertainties in anticipation of a future full trial including recruitment rates, acceptability of the BCPPA intervention, whether the intervention could be delivered as intended and an appropriate primary outcome measure.

- What are the key feasibility findings?

The BCPPA intervention was acceptable to people with PPA and their communication partners. Treatment fidelity was high (87.2%) and an appropriate measure identified (The Aphasia Impact Questionnaire).

- What are the implications of the feasibility findings for the design of the main study?

Though fulfilling continuation criteria, this research has identified unanswered questions that should be addressed prior to proceeding to a full trial.

## Background

Primary progressive aphasia (PPA) describes a group of language-led dementias underpinned by frontotemporal lobar degeneration or Alzheimer’s disease [1]. People with PPA experience a gradual dissolution of language as the leading symptom, with additional cognitive and behavioural symptoms typically developing later [2, 3]. There are three internationally recognised PPA variants; semantic (svPPA), nonfluent (nfvPPA) and logopenic (lvPPA) each presenting with differing language profiles [2, 3]. SvPPA is described as a gradual dissolution of semantic knowledge, with associated difficulties in using and understanding words, particularly nouns. People with nfvPPA experience effortful, halting speech (speech apraxia) and/or difficulties in understanding and using grammar. Finally, lvPPA causes difficulties in word retrieval and verbal short-term memory, meaning that people hesitate frequently, produce speech sound errors and present with difficulties in processing complex sentences [1].

Though this rare dementia constitutes only a small proportion of the total dementia burden, with an estimated 3000 people living with a diagnosis in the UK [4]. PPA has a disproportionate impact on social interaction and therefore interpersonal relationships. Whilst PPA is an extreme dementia phenotype, research on interventions for PPA have implications for managing common communication difficulties experienced by the wider population living with dementia.

Despite the dominance of language impairments in PPA, research into speech and language therapy for PPA is limited. Most of the work in this relatively sparse literature has centred around impairment-focused interventions that aim to maintain and improve access to personally relevant words and phrases [5]. The ultimate target in speech and language therapy is successful communication in everyday life. Yet, studies investigating functional interventions, which aim to support a person to execute an activity or participate in a life situation, have only recently been described in the research literature on PPA [6].

One such intervention, communication partner training (CPT) aims to change conversation behaviours in both the individual with a communication difficulty and their communication partner (CP), commonly a family member. A person and their CP are often referred to together as a dyad. Investigations of the impact of PPA on conversations demonstrate why this is important: strategies adopted by a person with PPA and their CP can result in conversation breakdown, or conversely, can be facilitative [7, 8] Speech and language researchers [9, 10] have previously advocated CPT as an important treatment approach for improving conversations between people with PPA and their families. UK speech and language therapists (SLTs) are in agreement with this position; a recent survey demonstrated that CPT approaches are prioritised above word relearning interventions in the clinical field of speech and language therapy for PPA [11]. To date only a few individual case studies of CPT for PPA exist in the published literature, and whilst Murray [12] and Wong et al. [13] report promising results, these studies lack rigour, both in the study design and reporting of the intervention.

CPT has a growing evidence base in stroke aphasia [14, 15] and traumatic brain injury [16] and can prevent the evolution of poor mental health outcomes for people with stroke aphasia [17]. It is important to note, however, that these programmes are not designed to meet the needs of people with progressive communication difficulties [18]. Communication strategies taught may not continue to be useful, and the ability of the person with PPA to engage with strategy use may alter. In response to this gap in the evidence base, the authors coproduced with key stakeholders a CPT program for people with PPA and their CPs entitled Better Conversations with PPA (BCPPA) [19, 20]. Based on an approach to CPT developed for stroke aphasia, Better Conversations with Aphasia (BCA [14]), and informed by the Medical Research Council guidance on developing complex interventions [21], BCPPA is the first manualised CPT intervention for PPA. For a full description of the coproduction of the intervention, see Volkmer et al [20].

## Study aim

Having developed the intervention, the primary aim of this study was to pilot the BCPPA program compared to a no speech and language therapy treatment control group at participating sites to establish for a main trial whether BCPPA can be delivered as intended in an NHS setting. Specifically, the aim was to establish:

1. Predicted patient recruitment and retention rates
2. Refinement of inclusion criteria
3. Acceptability of the intervention
4. An assessment of BCPPA treatment fidelity
5. The most appropriate primary outcome measure
6. A sample size calculation

This trial was retrospectively registered on 28/02/2018, ISRCTN10148247 https://doi.org/10.1186/ISRCTN10148247. The trial conforms to the CONSORT (Consolidated Standards of Reporting Trials) guidelines [22], see CONSORT checklist in supplementary materials, and the SPIRIT (Standard Protocol Items: Recommendations for Interventional Trials) statement [23].

## Methods

### Design

This was a single-masked, randomised controlled pilot study of BCPPA intervention program versus no speech and language therapy treatment, employing a randomisation ratio of 1:1. As part of, and alongside, the pilot RCT, a process evaluation was conducted. Participants were involved for a total of 6 weeks: pre-intervention measures (week 1); intervention/control (weeks 2–5); post-intervention measures (week 6). This study was granted ethical approval by London-Camden and Kings Cross Research Ethics Committee (reference: 17/LO/0357, received 26/04/2017). After initially identifying three sites, a further eight sites were added incrementally due to difficulties in participant recruitment related to staffing and service restructuring. Minor amendments to the study protocol were approved by the London-Camden and Kings Cross Research Ethics Committee. This report has followed the CONSORT (Consolidated Standards of Reporting Trials) guidelines 2010 statement: extension for randomised pilot and feasibility trials [22].

### Eligibility criteria

Participants were eligible if they had a diagnosis or possible diagnosis of any syndrome of PPA (in line with international consensus criteria [2]), were aged ≥ 18 years and had a conversation partner (CP) available who consented to participate in the study. Full details of inclusion and exclusion criteria are given in the published trial protocol [19].

### Setting

Eleven participating NHS sites were located across England and Wales. Table 1 provides an overview of the characteristics of the participating sites. Local collaborators (SLTs) at these sites recruited participants, obtained consent, completed pre-intervention measures and delivered the BCPPA intervention.

**Table 1:** Characteristics of sites participating in the BCPPA pilot RCT

Characteristics of sites number of sites (*n* = 11)	
Type of healthcare service:	
General hospital and community health service	3
Specialist neurology hospital	1
Community health service	2
Mental health service	4
Mental and physical health service	1
Area served by the healthcare service:	1
National centre	5
Urban Regional	5
Number of local collaborators trained per site:	
1 SLT trained	3
2 SLTs trained	1
3 SLTs trained	4
4 SLTs trained	1
5 SLTs trained	2
Setting where patients with PPA are seen:	
Outpatient	2
Community	5
Both outpatient and community	4

### Identification and recruitment of participants

Local collaborators were asked to identify eligible patients referred to their service, between 30/11/2017 and 31/12/2020 and to invite them to participate. Participants’ clinical care was not affected by a decision whether or not to participate. Accessible information sheets were provided at least 48 h before informed consent was obtained (see consent flowchart in published protocol [19]). Local collaborators completed a log to record the number of patients referred to their service with a diagnosis of PPA who did not meet the inclusion criteria. They also recorded the number of potential participants who were eligible, but who declined to participate in the study and their reasons why, if provided. This information was used to supplement recruitment and retention data.

### Randomisation

Randomisation was conducted by a member of the team not involved in data collection or intervention delivery using a random number generator and stratified by site using blocks of four to balance across the BCPPA intervention and no speech and language therapy treatment control groups within each site. Block sizes were not disclosed to local collaborators. Local collaborators were informed of participant group allocation by telephone by the first author after pre-intervention outcome measures had been completed.

### Sample size

In line with guidance on conducting pilot studies, one of the aims of the study was to predict recruitment and retention rates for a future full trial [24, 25]. As there were no data available to estimate a sample size for the current study, the recruitment of participants was dealt with pragmatically, based on logistics, resources and time [26]. Following discussions with clinicians at the primary research site, it was originally estimated that it would be possible to recruit 42 participants across three research sites in England over an 18-month period. Recruitment was reviewed at 2-month intervals during the study and the strategy amended as necessary. Recruitment was slower than anticipated and complicated by changes in staffing and service delivery models within the local speech and language therapy departments, as well as the onset of the COVID-19 Pandemic. As a result, further research sites were identified across England and Wales, and the recruitment period for the study was extended by 24 months. Given the ongoing nature of the COVID-19-related restrictions, the research team decided to close the study, prior to the identified extension date, with a sample size of 18 participants.

### Description of the intervention

#### BCPPA intervention

BCPPA provides a manual for SLTs to deliver four 1-h sessions of a CPT intervention for people with PPA and their CPs. Local collaborators were trained to deliver the BCPPA program by the first author and were given access to an online BCPPA manual and training resource. No prior knowledge or experience of PPA was required. Table 2 presents an overview of training content. The development of the intervention is described in detail in Volkmer et al. [20] and an overview of the four BCPPA sessions and their aims is presented in Supplementary Material using the Template for Intervention Description and Replication (TIDieR) checklist [27] in the published protocol [19]. Participant dyads video recorded four conversation samples pre-intervention, which provided short clips for video feedback during intervention sessions.

**Table 2:** Overview of training of local collaborators

Training goals	Pre-training work	Training activities
To be able to: • Identify potential participants who meet the study inclusion criteria • Collect conversation samples to inform intervention delivery Analyse conversation to inform and plan for delivery of BCPPA • Deliver four BCPPA sessions using manual, session plans and handouts	Local collaborators were given access to the online BCPPA manual and training resource and sent reading materials from the Study Training Pack, e.g. example completed session plans	• Discuss inclusion/exclusion criteria with vignettes to problem solve Discuss and practice video recording conversation samples • Observe sample video recordings and identify barriers and facilitators to conversation with a view to planning intervention • Discuss and practice preparing for therapy session using session plans Role play introduction of therapy tasks Observe and reflect on video examples of BCPPA being delivered

#### No speech and language therapy treatment

Results of a UK-wide survey of SLTs highlighted that there is no standard clinical speech and language treatment for people with PPA [28]; therefore, a no treatment control group was selected. In addition, this survey highlighted the difficulties for many people with PPA in the UK in accessing speech and language therapy, thus a no treatment control group is not dissimilar to standard clinical care. Word relearning might seem to be an obvious alternative control, given the amount of research evidence in this area. However, as Cadorio et al. [29] highlight, people with different PPA syndromes require different word relearning interventions, thus this approach would lack homogeneity as a control condition. An alternative more homogenous social control would be challenging, as it would be difficult to disentangle the active ingredients of CPT from a typical attention control group, e.g. activities to promote social interaction (see Palmer et al. [30]). The use of a no treatment control is in line with other RCTs for people with dementia (Cognitive Stimulation Therapy [31]) and stroke aphasia trials [32].

Those participants assigned to the no speech and language therapy treatment condition received usual healthcare provision (anticipated to include neurology, GP reviews, and allied health input such as physiotherapy).

The period of no speech and language therapy was 6 weeks (4 weeks when the treatment group received BCPPA, and 1 week each when both groups completed pre- and post-intervention assessments). After 6 weeks, the participants allocated to the no speech and language therapy treatment group resumed all aspects of local speech and language therapy provision that were available without further interruption.

### Assessment of acceptability of the intervention

Anonymous feedback was collected to ensure BCPPA was acceptable to people with PPA, their CPs and local collaborators. Accessible feedback forms were given to participants with PPA and their CPs at every intervention session, to be completed jointly by the dyad and returned anonymously in pre-stamped addressed envelopes directly to the research team. Feedback forms comprised 13 questions, including closed questions (multiple choice and ratings) for speed and ease of response and open questions to elicit additional information (see Supplementary Materials). Local collaborators were asked to include feedback on acceptability as part of a fidelity questionnaire, completed after every intervention session.

### Assessment of treatment fidelity

In this study, two aspects of treatment fidelity were assessed—fidelity of delivery and enactment [33]. To allow for investigation of fidelity of delivery, all local collaborators were asked to video or audio record themselves delivering BCPPA intervention sessions with all participant dyads. In line with recommendations for measuring treatment fidelity [34, 35], a random sample of 20% of these session recordings were chosen for analysis, using a random list generator. Using methods for developing measures of fidelity for complex interventions [31], data were analysed using a fidelity checklist and a coding manual. These were developed from the BCPPA intervention manual by two junior researchers (UCL student SLTs as part of their MSc dissertations), supervised by AV, SB and HW. Fidelity analysis identified fidelity scores for both standardised components, identified as compulsory, and tailored components, identified as optional for delivery of the intervention. Details of the development of the fidelity checklist and coding manual and the assessment of enactment are published elsewhere [36]. Although fidelity data are sparse for speech and language intervention trials, processes such as those deployed in our study have been shown to achieve an average 80–100% fidelity [37–40]. Thus, we selected 80% as the minimum criterion for fidelity. The development of a coding spreadsheet with accompanying guidelines to measure enactment are also described in Volkmer et al. [36] and are not further reported on in this paper.

### Masking

Post-intervention measures were collected by pairs of junior researchers (student SLTs at UCL), who were crucially masked to group allocation. Participants and CPs were asked not to reveal their group allocation to the junior researchers during the post-intervention outcomes session. They were reminded of this prior to their appointment, by letter or phone, and verbally at the start of the session.

### Pre- and post-intervention measures

To identify the appropriate outcome measure for a future large-scale trial various measures were trialled. In order to objectively confirm the PPA variant, a language assessment was included. Given the aim of the BCPPA intervention was to reduce the impact of PPA-related communication difficulties and improve quality of life, a range of quality of life measures were identified from the stroke aphasia and dementia literature that were validated and widely used in clinical practice (a full description of the outcome measures can be found in the study protocol; Volkmer et al. [19]). Additionally, based on the conversation measure used in the CPT evaluation study of Best et al. [14], four 10-min baseline conversation samples were video recorded. The first (sample 1a) was recorded in week 1 with the assessor present but not in the same room (to avoid overt observer paradox). At this session, participants were trained to independently use an iPad to video record two further conversation samples independently at home (2i and 3i). The final baseline recording (4a) was made in week 1 at the pre-intervention assessment session. Four post-intervention conversation samples were recorded, one in week 6 (5a, assessor present but not in the room) and three independently recorded at home in week 7 (samples 6i, 7i and 8i). A conversation topic list was provided to support this process should the dyad require it. Conversation samples were analysed by junior researchers, masked as to whether recordings were recorded pre- or post-intervention. Goals set in therapy were operationalised as observable conversation behaviours and coded and counted across video recorded conversation samples to identify change in the frequency of dyad behaviours pre-post-intervention.

Outcome measures listed in Table 3 were completed in week 1 by the local collaborator and in week 6 by the pairs of masked junior researchers. Participant dyads randomised to the BCPPA intervention arm also completed Goal Attainment Scaling (GAS [41]) during the intervention itself, both to determine specific individualised targets for the intervention, and to measure change in these targets. With support from the local collaborator, participant dyads identified goals during session 2 and weighted the goals according to their importance and likelihood of being achieved. At the end of therapy, the goals were reviewed by the participant dyads with the support of local collaborators and an outcome score assigned. Table 4 summarises the schedule of pre- and post-intervention measures.

**Table 3:** Outcome measures collected pre- and post-intervention for the BCPPA pilot study

Respondent	Measure
PwPPA	Aphasia Impact Questionnaire 21 (AIQ-21 [42])
	Dementia Quality of Life Measure (DEMQOL [43])
	Communication Confidence Rating Scale for Aphasia (CCRSA [44])
CP	Perceived Stress Scale (PSS [45])
	Zarit Burden Scale [46]
Dyad	Four 10-min video recorded everyday conversations

*PwPPW* Person with primary progressive aphasia, *CP* Communication Partner

**Table 4:** Schedule of pre- and post-intervention measures

	Consent session			Pre-intervention Assessment (1 week)	Treatment/ Control (4 weeks)				Post-intervention Assessment (1 week)	Final data collection		
Week	0			1	2	3	4	5	6	7		
Video recording of everyday conversation	1a	2i	3i	4a					5a	6i	7i	8i

Video recording of everyday conversation: a—assessor present but not in room; i—independent home recording

### Data management

Participant dyads were given a unique number which was used to store all information on diagnosis, medical and social history and on all paperwork including assessment score sheets, in the names of video files, and in all subsequent analysis documents and publications. At each NHS site, the local collaborator stored a list of the participant names and their unique identifiers (required to conduct the remote randomisation procedure) in a locked cabinet. Each list only contained the names of participants based at the relevant site.

Participant dyads consented to be video recorded in conversation for the purposes of outcome measurement, and to provide clips for video feedback during intervention sessions. Only the author, her supervisors and the junior researchers had access to the video data set. Two levels of data were collected: (i) non-anonymisable (facial expression) data, these were only for analysis purposes or teaching presentations with the participants’ explicit consent, but not for publication, (ii) anonymisable (transcripts, audio), for potential publication. Participants’ faces are fully visible in these video recordings as facial expression forms a significant part of natural human communication, the focus of this intervention. As a result, whilst confidentiality could be guaranteed in the use of footage for presentations, the preservation of anonymity was not possible. Judicious selection of recordings minimised this risk (e.g. footage where personal details were discussed was not used and names were blanked out of the audio stream). Participants and their CPs were asked whether they were willing to accept the possibility of being recognised by consenting to use of their video data for presentations. If not, they could choose to opt out of use of their data in this way whilst remaining part of the study. Where conversation data were transcribed for evaluation, they were anonymised through the use of pseudonyms for all named people and places.

### Data analysis

Criteria to proceed to a full trial were identified in advance of the study as:

- Patients and local collaborators report generally positive views about the acceptability of randomisation, and of the intervention, as determined by evaluation of feedback forms;
- Local collaborator intervention fidelity rate is at least 80%;
- A suitable sensitive outcome measure is determined, and sample size estimated.

Descriptive statistics were used to report recruitment, attendance and attrition data including reasons for dropout. Participant dyad feedback and fidelity data were reported using descriptive statistics and narrative data.

Outcome measure data were entered into a database and analysed using the Statistical Package for the Social Sciences version 25 [47] and the G*Power 3.1 software [48]. The guide to using GAS provided information on calculating baseline and attainment scores [38]. Data from GAS scores were entered into an Excel spreadsheet, the weighting was calculated by multiplying the importance rating by the difficulty rating. The extent to which each dyad’s goals were attained was calculated using a standardised formula: $\mathrm{GAS}\;\mathrm{score}=\lbrack50+10\Sigma(w_ix_i)\rbrack/\lbrack0.7\Sigma w_i0.3(\Sigma w_i)\rbrack$, where *w*_*i*_ = weight assigned to the goal and *x*_*i*_ = the attained score for the goal. In this study, all goals were given a baseline rating of − 1 (in line with guidelines for using GAS in research) and both a baseline and attainment GAS score calculated using the above formula.

Video recordings of everyday conversations for enactment analysis were divided between junior researchers for transcription and coding (AB, TC, CB, CR, NT and MC). Videos were allocated using an online random list generator. Transcripts were pseudonymised and researchers masked to the allocation (BCPPA treatment or control) and schedule of recording (pre- or post-intervention). Researchers independently coded the recordings they had transcribed, remaining masked throughout this process in order to prevent any bias. In order to code the video recordings, a coding spreadsheet and accompanying guidelines were developed (see Volkmer et al [36]), informed by methodology developed by Best et al. [14] to measure communication outcomes. Each participant’s goals were converted into an observable behaviour, and transcribed video recorded conversations analysed to identify and count these behaviours. Behaviours were categorised as to whether they described a facilitator behaviour, that enhanced the progressivity of conversation, or a barrier behaviour, that prevented progressivity, resulting in temporary breakdown of conversation [49]. Once unmasked, descriptive statistics was used to report baseline and attainment data in behaviours that correspond with goals set by each participant.

A minimally clinically important difference (MCID) is defined as the smallest change between two scores that is subjectively meaningful to patients [50]. It was not possible to use this approach to inform the exploration of the most suitably sensitive outcome measure as a MCID has not previously been established for any of the measures used in this study. Consequently, a sample size calculation was conducted. To inform a sample size calculation the mean pre- and post-intervention scores, and a mean change score, 80% confidence intervals (CI) and standard deviations were calculated for each measure. The mean change scores and standard deviations were entered into G*Power software and an effect size calculated. This then informed a two-tailed sample size calculation for each measure.

It is not considered appropriate to report effectiveness calculations for pilot studies as they are considered underpowered and unrepresentative [22]. Therefore, results were not tested for statistical significance and only descriptive statistics were reported, in line with current CONSORT guidance on conducting a pilot study [22].

## Results

The data reported here were collected over a 23-month period from November 2017 to December 2020.

### Screening, recruitment and retention

Of the 11 research sites participating in the study, four had to pause their involvement in the study on one occasion, and one site had to pause involvement on two occasions and eventually discontinued participation. Of the 31 SLTs trained as local collaborators eight left their positions or were unable to continue as local collaborators. All remaining sites paused involvement due to the COVID-19 pandemic from March 2020 to October 2020. Four sites volunteered to continue remotely but were unable to recruit any participants. No new participants were recruited after January 2020 and the study was closed in December 2020.

**Fig. 1:**
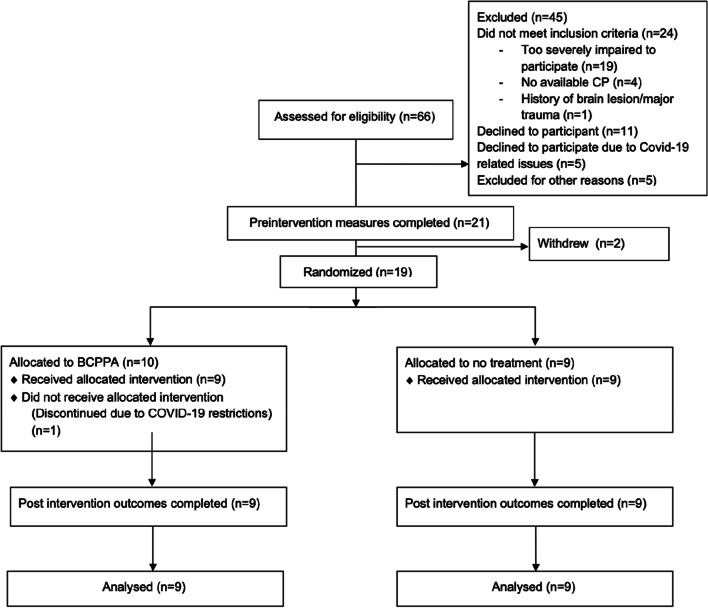
Participant flow chart (CONSORT flow chart) describing recruitment and retention of participants in BCPPA pilot study

Sixty-six people were screened for potential inclusion in the study. Of these potential participants, 45 were excluded: 24 did not meet the inclusion criteria, 11 declined to participate, despite being eligible, five declined to participate due to COVID-19 related issues, and five were excluded for other reasons (see the CONSORT flow chart in Fig. 1). Twenty-one participant dyads, who were deemed eligible and consented to participate in the study, were recruited from seven research sites. Of these, two withdrew from the study immediately after the pre-assessment process reporting that they found the assessment process too challenging. Nineteen participant dyads were randomised, one was discontinued at the onset of the nationwide restrictions related to COVID-19 pandemic and the remaining 18 completed the study. Junior researchers completed the post-intervention assessment with all 18 participant dyads and remained masked to randomisation for 15 of these. On two occasions, the participant dyads revealed their allocation prior to assessment.

### Demographic and clinical characteristics of participant dyads who completed the study

The characteristics and demographic information of the participants with PPA and their CPs who completed the study are presented in Table 5. All participants with PPA and their CPs spoke English as their language of daily use. The intervention group comprised four men and five women with PPA who had an average age of 72.1 years (range 57–85 years). Seven of the CPs were spouses, and two were adult children, with an average age of 64.6 years (ranging from 34 to 80 years). The no treatment control group comprised four men and five women with PPA who had an average age of 71.3 years (ranging from 63 to 85 years). Eight of the CPs were spouses, and one a close friend, with an average age of 71.6 years (ranging from 69 to 85 years).

**Table 5:** Demographic and clinical characteristics of participant dyads who completed the study

	**BCPPA**	**CP (BCPPA)**	**No treatment**	**CP (No treatment)**
	*n*=9	*n*=9	*n*=9	*n*=9
**Age (years)**	72.1	64.6	71.3	71.6
**Age (range)**	57-85	34-80	63-85	69-85
**Gender (m:f)**	4:5	4:5	4:5	4:5
**nfv PPA (n)**	4	-	2	-
**lvPPA (n)**	3	-	5	-
**svPPA (n)**	0	-	1	-
**Mixed PPA (n)**	2	-	1	-
**Time since symptom onset (mean and range in months)**	37.3 (27-67)		46.9 (20-84)	
**Time since diagnosis (mean and range in months)**	18.1 (5-36)		20.4 (8-72)	
**Education**	Secondary: 5 Tertiary: 4	Secondary: 2 Tertiary: 2 Not known: 5	Secondary: 6 Tertiary: 2 Not known: 1	Secondary: 3 Tertiary: 2 Not known: 4
**Occupational status (Recorded prior or current occupation)**	Retired: 8 Working: 1 (1=manual, 8=intermediate/high managerial)	Retired: 2 Working: 3 (2=manual, 3=intermediate/high managerial) Not known: 4	Retired: 9 (2=manual, 5=intermediate/high managerial)	Retired: 3 Working: 2 (2=manual, 3=intermediate/high managerial) Not known: 4
**Comprehensive Aphasia Test **[51] **mean sand range cores on**				
Comprehension of spoken language (max score 66)	53 (19-64)		46.6 (21-62)	
Repetition score (max 74)	47.4 (5-71)		49 (25-71)	
Naming objects (max score 48)	35.3 (0-48)		23 (0-48)	
Picture description	26.2 (3-50)		23.1 (1-50)	

*BCPPA* Better Conversations with Primary progressive Aphasia, *CP* Communication Partner, *m* male, *f* female, *nfvppa* non fluent agrammatic variant primary progressive aphasia, *lvPPA* logopenic variant primary progressive aphasia, *svPPA* semantic variant primary progressive aphasia

Of the 18 participants, eleven had been diagnosed with a PPA variant and seven with no specific variant specified. Examination of pre-intervention language test data [52] was used to confirm diagnosis of PPA variant in line with the Gorno-Tempini et al. [2] internationally agreed diagnostic criteria. The data collected confirmed the eleven pre-existing diagnoses and of the seven participants with no specific variant four were diagnosed with a specific PPA variant, whilst the remaining three were judged to present with symptoms consistent with mixed PPA. Of the four given new diagnoses, three were given a diagnosis of lvPPA (as there were no signs of speech apraxia, but difficulties in digit span, sentence repetition and word retrieval in the presence of relatively spared comprehension of single words) and one participant was given a diagnosis of nfvPPA (due to the presence of apraxia and agrammatism with relatively intact comprehension). Of the nine participants randomised to the BCPPA treatment arm, four had a diagnosis of nfvPPA, three lvPPA and two mixed PPA. In the control arm, two had nfvPPA, five lvPPA, one svPPA and one mixed PPA.

All nine dyads with PPA who completed the BCPPA intervention were included in the analysis. The DEMQOL and CCRSA data from one participant randomised to the no treatment control group were excluded due to significant fatigue during post-intervention assessment, resulting in scores at floor. This was not consistent with performance at baseline, nor with other measures taken during the post-intervention assessment session.

### Acceptability of randomisation

#### Acceptability to participant dyads

All dyads who participated in the BCPPA intervention arm completed and returned anonymised feedback forms following each intervention session. The following provides a more in-depth analysis of the feedback received:

##### Explanations, format, delivery and expectations of the intervention

**Fig. 2:**
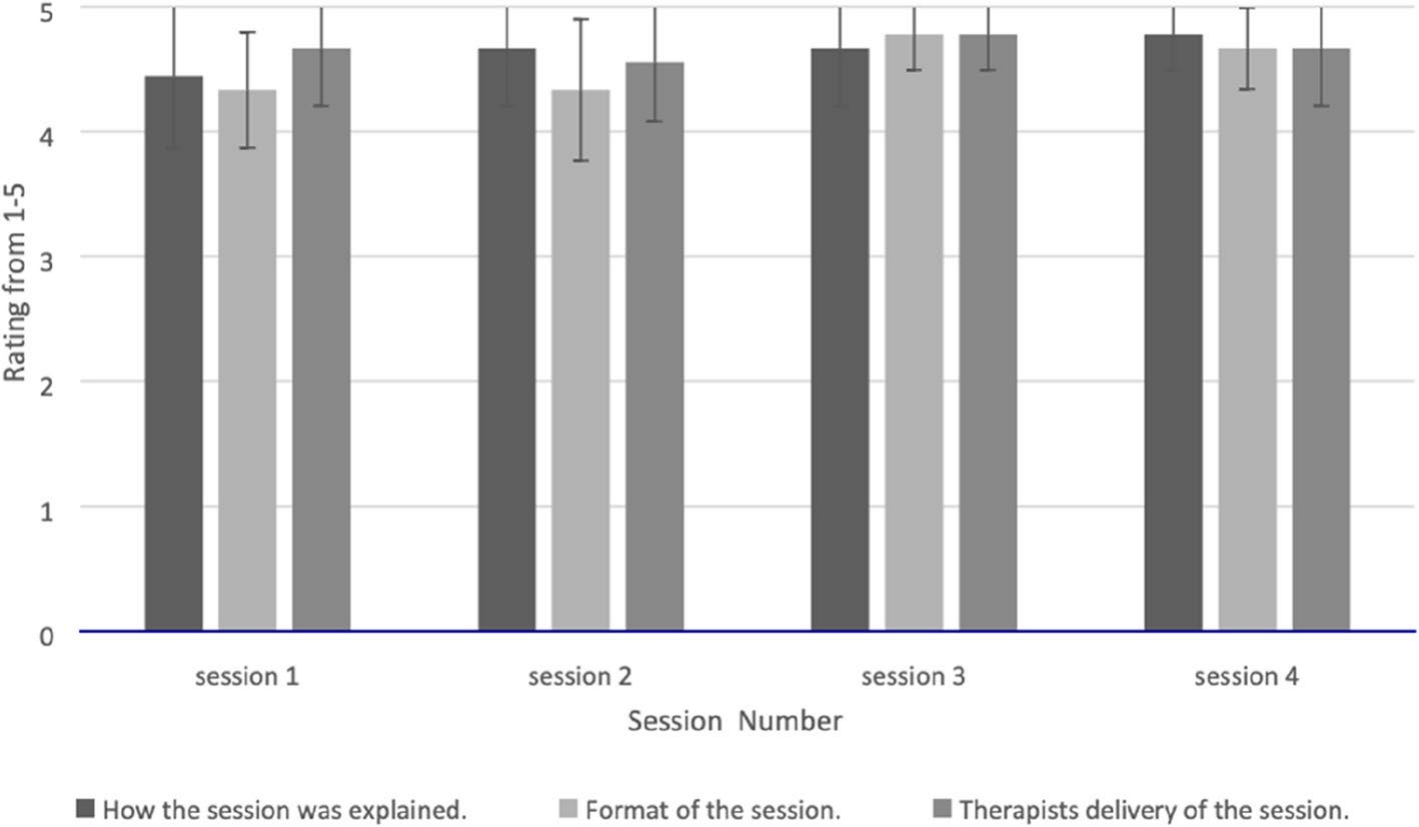
Participant dyads’ mean ratings of the explanation, format and delivery of BCPPA intervention sessions

Ratings of intervention sessions relating to explanations given, format and delivery increased as therapy progressed, with session 1 scoring an average of 4.5 compared to an average of 4.7 for session 4 (see Fig. 2). Similarly, the participant dyads’ expectations of intervention changed over time. At the start, only four of nine dyads reported that session 1 was what they had expected, but by session 4 all dyads reported the session met their expectations. Only one suggestion was made of an addition to the intervention: “to be told how to find alternative words when stuck”.

##### Usefulness of the intervention

Dyads considered on average 92% of the intervention were useful. Two dyads commented specifically on the utility of watching the video clips of themselves and of the handouts. Video feedback was considered useful throughout the intervention by 69% of dyads, although one respondent with PPA expressed a preference not to see themselves on video.

##### Improvements as a result of the intervention

**Fig. 3:**
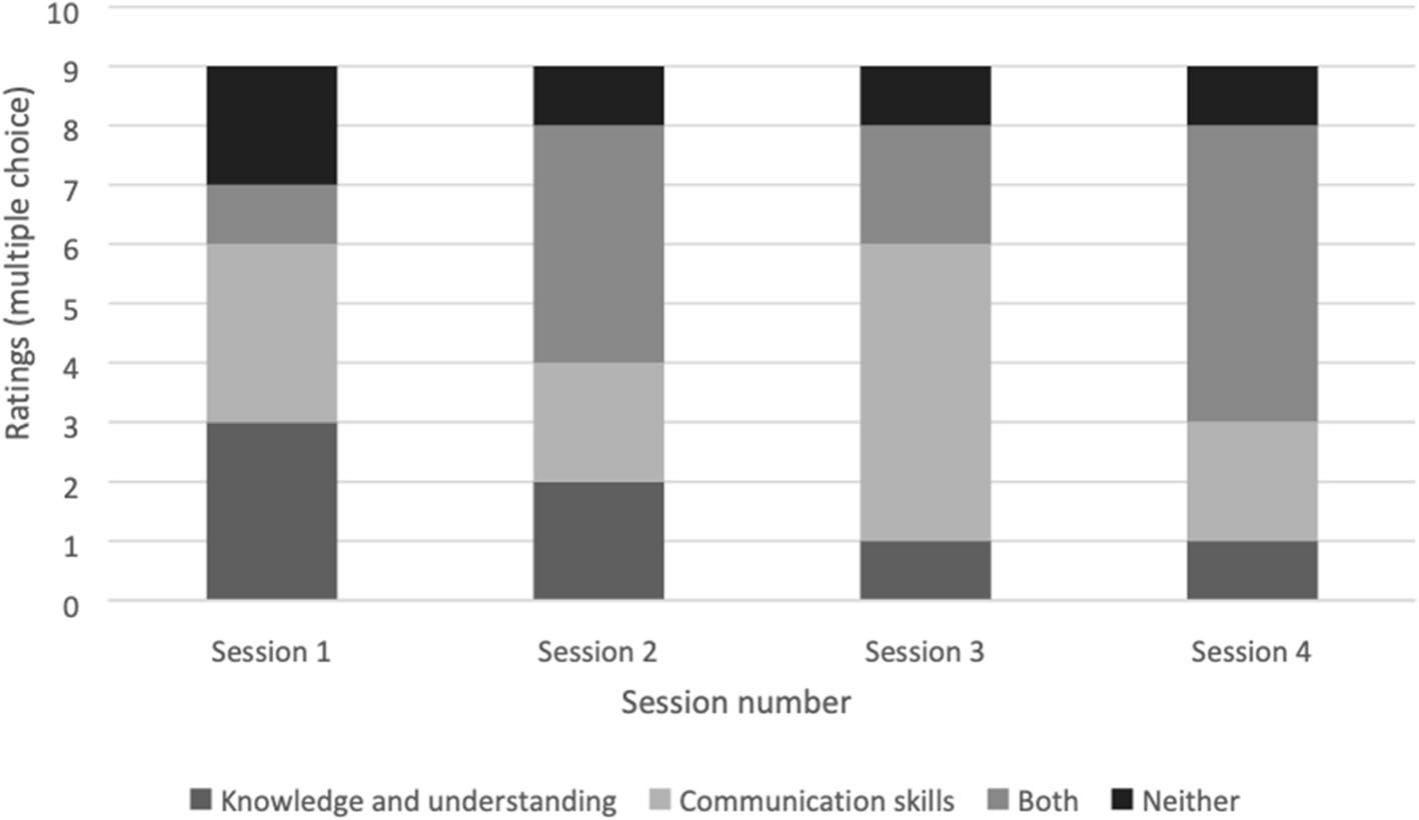
Participant dyads’ ratings of whether BCPPA intervention sessions improved their (i) knowledge and understanding of PPA and (ii) communication skills

Dyads were asked whether each session improved their (i) knowledge and understanding of PPA and (ii) communication skills. By session 4, 56% of participant dyads rated both domains as improved, and only 11% rated no improvement in either (see Fig. 3).

**Fig. 4:**
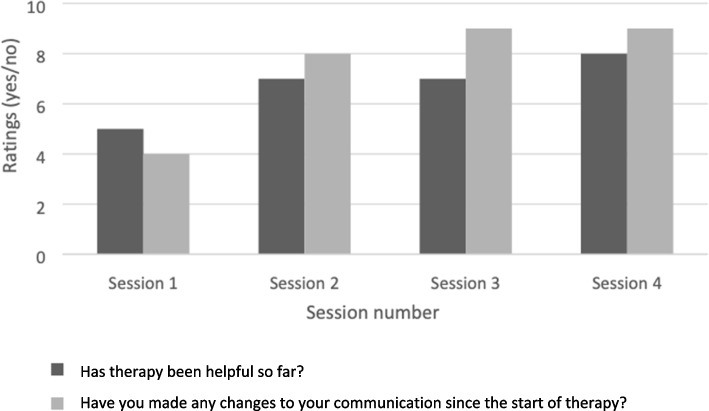
Participant dyad’s ratings of whether BCPPA intervention sessions were helpful and whether they had made any change in their communication

Participant dyads were asked whether therapy was helpful, and if they had made changes in their communication since starting therapy. After session 1, 56% reported it was helpful and 44% that they had made communication changes. By the end of therapy, 89% reported that therapy was helpful and 100% that they had made changes in their everyday dyadic communication (see Fig. 4).

#### Acceptability to local collaborators

Local collaborators who delivered the BCPPA intervention completed a fidelity questionnaire for every participant dyad they treated. Table 6 summarises data on aims met, session length, setting, and confidence.

**Table 6:** Local collaborators’ responses to fidelity questionnaire items on aims met, session length, setting and confidence

	Session 1	Session 2	Session 3	Session 4
Number of aims met	100%	100%	100%	100%
Mean length of session in minutes (range)	58 (40–70)	60 (60)	61 (55–65)	62 (60–75)
Setting delivered	7 = at their home 2 = hospital outpatients	7 = at their home 2 = hospital outpatients	7 = at their home 2 = hospital outpatients	7 = at their home 2 = hospital outpatients
Local collaborators rating of their own confidence in delivering the session (very confident, confident, somewhat confident, not at all)	Very confident: 1 Confident: 6 Somewhat confident: 2	Confident: 6 Somewhat confident: 3	Very confident: 1 Confident: 6 Somewhat confident: 2	Very confident: 1 Confident: 7 Somewhat confident: 1

**Fig. 5:**
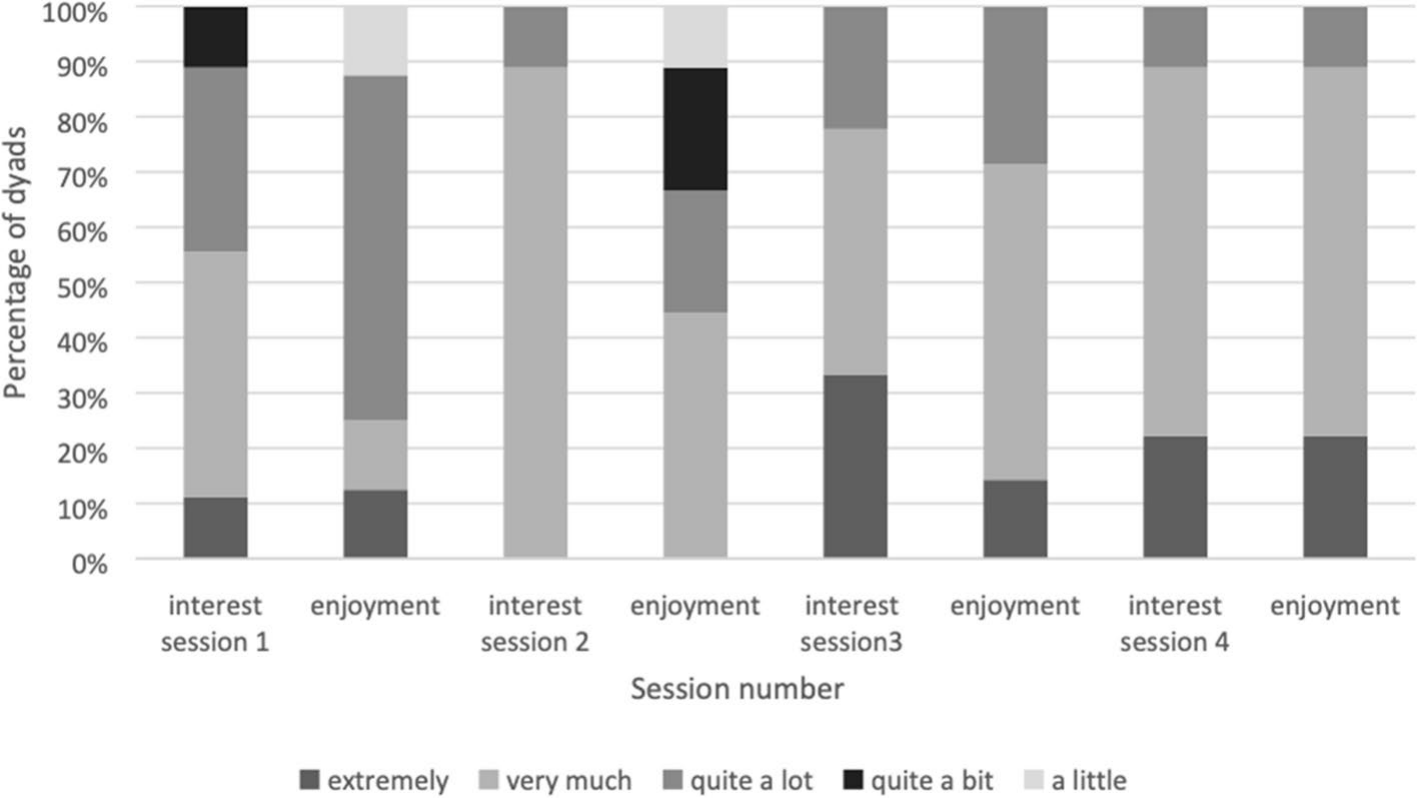
Local collaborators ratings of dyads’ interest and enjoyment of the BCPPA intervention sessions

Local collaborators were asked to rate how interesting and enjoyable the dyad found the sessions on a 5-point scale (a little, quite a bit, quite a lot, very much or extremely). Ratings increased as the intervention progressed. In terms of interest of dyads, local collaborators rated 55% as finding session 1 “very much” or “extremely” interesting. In terms of enjoyment, local collaborators rated 22% of dyads as having enjoyed it “very much” or “extremely” (see Fig. 5). By session 4, 89% of dyads were rated by local collaborators on the top two points of both the interest and enjoyment scales (“very much” or “extremely”).

Local collaborators were invited to make free text comments on the dyads’ enjoyment and interest during the intervention. One local collaborator reported that a CP found the discussion and handouts “a bit infantile” in session 1. Another explained that it was difficult to focus discussion on the CP’s communication skills as well as those of the person with PPA in session 2. One dyad reportedly “really enjoyed the practice tasks” in session 3, and another local collaborator felt that the conversations between the dyad started to feel more natural at this point. Following session 4, two local collaborators reported it had been difficult for a CP to discuss future deterioration.

Local collaborators were also invited to make general comments. One reported a preference for delivering the intervention in a different order than prescribed in the session plans, namely presenting more of the video feedback (session 2) before giving the information (session 1) on how conversation works. Another two commented on technical difficulties experienced when showing video clips. Three commented on session 2 requiring more preparation time than session 1. Following session 4, two local collaborators suggested the dyads they worked with could have benefitted from further practice. In contrast, another reported the dyad they worked with could have had one less session.

#### Summary

Dyads rated explanation, format and delivery of the intervention highly. They rated the intervention as generally useful, and after the last session, all dyads rated that they had made a change as a consequence of the intervention.

The majority of local collaborators reported feeling confident in delivering the intervention. They reported an increase in the interest and enjoyment of the dyads as sessions progressed. They also reported suggestions for refinement of the intervention.

### BCPPA treatment fidelity

Treatment fidelity was 87.2% for the standardised components of the BCPPA intervention. For tailored components, it was 63.8%. Average fidelity scores for each of the four sessions, following rater discussion and agreement are presented in Table 7. Inter-rater reliability across the eight observed sessions was 90.74% (range = 80.95–100%) prior to discussion and agreement between raters. A detailed breakdown of fidelity ratings and enactment of the intervention are reported in the paper describing the full fidelity methodology developed for this study [33].

**Table 7:** Treatment fidelity per session, across standardised and tailored components of the BCPPA program

	Standardised components		Tailored components	
	Average score / maximum score	%	Average score	%
Session 1	16.5 /18	91.67%	14 /14	100%
Session 2	20 / 26	76.92%	9.5 / 16	59.38%
Session 3	15 / 16	93.75%	17.5 / 30	58.33%
Session 4	19 / 22	86.36%	10 / 20	50%
Total percentage adherence:		87.2%		63.8%

In line with recommendations for measuring treatment fidelity, this table represents data from a random sample of 20% of sessions, selected using a random list generator to identify two dyads

#### Assessment of outcome measures for a full-scale trial

The PwPPA completed the AIQ-21, DEMQOL and CCRSA, the CP completed the PSS and Zarit Burden Scale. Feedback from local collaborators and junior researchers indicated a preference for the AIQ-21 [42] over other measures. They reported it was the most meaningful and practicable measure to complete with participants as it linked most closely to the purpose of the intervention (to reduce the impact of PPA on a person’s conversation), and the images made it most accessible. This was the only measure completed with people with PPA where data from all participants could be included (due to fatigue resulting in increased cognitive impairment one participant’s data on the CCRSA and DEMQOL were excluded), emphasising its accessibility. Importantly, the AIQ-21 has been demonstrated to have statistically significant concurrent validity and good internal consistency, and the prototype has been demonstrated as sensitive to detecting change in people with stroke aphasia following a community intervention [42]. Thus the AIQ-21 presents a logical choice for a primary outcome measure.

The pre- and post-intervention scores, and the change scores from the five outcome measures are shown in Table 8. The AIQ-21 was completed by the PwPPA and results demonstrate a mean change score in the intended direction of − 3.33 (95% CI − 4.26, − 2.41) for the BCPPA intervention group, indicating a reduction in the impact of PPA. The mean change score of 2.78 (95% CI − 2.11, 7.45) for the no treatment control group indicates an increase in the impact of PPA. On closer examination of the AIQ-21 mean scores, it is apparent there is a large disparity between the BCPPA and the control group mean scores initially, such that the post-intervention score may be attributed to a regression to the mean (the phenomenon of scores being extreme on first measurement, and closer to the mean on second measurement) rather than the impact of the intervention. A future trial may be able to take account of this using a double baseline measure.

**Table 8:** Outcome measures completed with the PwPPA and the CP pre-intervention, post-intervention and change scores for the BCPPA intervention group and no treatment control group

Rater	Measure	BCPPA (*n* = 9)			No treatment control group (*n* = 8 for DEMQOL & CCRSA, *n* = 9 for all other measures		
		Pre-intervention	Post-intervention	Change score	Pre-intervention	Post-intervention	Change score
PwPPA	Aphasia Impact Questionnaire 21 (AIQ-21 [42])	19.78	16.56	− 3.33 (SD: 6.8)	11.22	14	2.78 (SD: 7.48)
	Dementia Quality of Life Measure (DEMQOL [43])	87.89	92.38	3.11 (SD: 9.65)	91.38	97.88	6.5 (SD: 11.21)
	Communication Confidence Rating Scale for Aphasia (CCRSA [44])	59.67	63.11	3.44 (SD: 13.4)	63.25	76	12.75 (SD: 11.12)
CP	Perceived Stress Scale (PSS [45])	13.66	12.78	− 0.89 (SD:2.37)	14.55	11.22	− 3.33 (SD:4.5)
	Zarit Burden Scale [46]	27.33	22.56	− 4.78 (SD: 7.66)	20.22	15	− 5.22 (SD:7.73)

*PwPPA* person with Primary Progressive Aphasia, *CP* Communication Partner, *BCPPA* Better Conversations with Primary Progressive Aphasia, *SD* standard deviation

Results from the DEMQOL, completed by the PwPPA, demonstrate a mean change score in the intended direction of 3.11 (95% CI − 3.19, 9.42) in the BCPPA intervention group, and 6.5 (95% CI − 1.27, 14.27) in the no treatment control group. This indicates both groups experienced an improvement in quality of life. The CCRSA was also completed by the PwPPA and results demonstrate a mean change score in the intended direction of 3.44 (95% CI − 5.31, 12.2) for the BCPPA intervention group and 12.75 (95% CI 5.04, 20.46) for the no treatment control group. This indicates both groups experienced an improvement in communication confidence.

The PSS was completed by the CP and results demonstrate a mean change score in the intended direction of − 0.89 (95% CI − 2.44, 0.66) for the BCPPA intervention group and − 3.33 (95% CI − 6.27, − 0.39) for the no treatment group, indicating both groups experienced a reduction in perceived stress. The Zarit Burden Scale was also completed by the CP and results demonstrate a mean change score in the intended direction of − 4.78 (95% CI − 9.78, 0.23) for the BCPPA intervention group and − 5.22 (95% CI − 10.27, − 0.17) for the no treatment control group, indicating both groups experienced a reduction in carer burden.

Goal Attainment Scaling (GAS) scores set and rated by participant dyads in the BCPPA intervention group indicate that of the 30 goals set, 20 were achieved more than expected, seven were achieved, two were achieved much more than expected and one goal was not achieved. The mean baseline score was 36.77 and the post-intervention mean attainment score was 59.13, resulting in a mean change score of 22.36 (95% CI 16.75, 27.95). Table 7 provides details of all goals and their attainment, as well as frequency of linked conversation behaviours observed via analysis of pre- and post-intervention video recorded conversations.

Each dyad set between two and five goals, resulting in a total of 30 goals. Each goal described a behaviour that participants wanted to consider and change, e.g. “To ask more questions”. Some goals comprised change in multiple behaviours, e.g. “To use more writing and drawing in conversation” meaning the 28 goals set described a total of 32 behaviours. Two goals set did not align with an observable conversation behaviour, instead targeting participant emotions and were excluded from this behaviour analysis. Of the 32 targeted behaviours, 18 demonstrate a change in the intended direction when comparing frequency of behaviours coded in pre- and post-intervention video recorded conversations. Seven demonstrate no change and 7 demonstrate change in the unintended direction (see Table 9).

**Table 9:** BCPPA goal attainment and frequency of linked conversation behaviours observed via analysis of pre- and post-intervention video recorded conversations

Dyad	Goals agreed by PwPPA, CP and the local collaborator *(NB: Each goal has been labelled to show who the goal belongs to)*	Achievement	Baseline score	Attainment score	Change score	Corresponding behaviour no. and description (see rating guidance for list of behaviours)	Pre-intervention frequency	Post- intervention frequency
2.01	To elaborate more (PwPPA)	A +	35.78	64.22^a^	28.44	1 (F)	8.33	6
	To wait or avoid finishing PwPPA’s sentences (CP)	A +				2 (B)	0	0
	To ask more questions (PwPPA)	A +				3 (F)	4	**7**^a^
	To ask more open questions (CP)	A +				4 (F)	0.7	0.7
3.01	To use gestures when encountering word finding difficulties (PwPPA)	A	35.81	58.9	23.09	19 (F)	0.33	**3**^a^
	To ask less test questions (CP)	A +				5 (B)	1.7	**0.7**^a^
	To give additional information around a person’s identity when unable to generate name (PwPPA)	A				6 (F)	0	**0.33**^a^
	To waiting before offering prompts to help (CP)	A +				7 (F)	0.3	**3**^a^
4.02	To use prompt card when needed to help facilitate conversations (PwPPA)	A +	38.76	63.3^a^	24.54	8 (F)	0	0
	To let PwPPA lead conversations (CP)	A + +				16 (F)	0	0
3.03	To pause when PwPPA gets stuck on a word and give a bit more time (CP)	A +	36.78	67.6^a^	30.82	7 (F)	3	0
	To use eye gaze to point, when stuck on a word, to indicate word/topic (PwPPA)	A +				24 (F)	0.33	0
	To use hands for gesture/mime to support talking (to prompt self & indicate to others) (PwPPA)	A + +				20 (F)	2.33	**12**^a^
1.01	To choose the topic (PwPPA)	A +	35.38	63.2^a^	27.82	9 (F)	3.67	3.67
	To describe or use gesture if I can’t think of the word (PwPPA)	A				11 (F) 19 (F)	0 0	0 0
	To ask single questions rather than either or questions (CP)	A +				12 (B) 14 (F)	10.3 0	**8.3**^a^ **1**^a^
	To use shorter sentences (CP)	A +				15 (F)	14.7	10.3
	To ask questions to prompt PwPPA to choose a topic (CP)	A +				16 (F)	7.7	**10**^a^
4.04	To use a key word then comment based on this, to try and expand responses (to avoid always saying yes / no or agreeing) (PwPPA)	A	37.64	43.1	5.46	17 (F)	18.67	16
	To use a gesture / signal when it’s the end of turn – difficult to know if finished OR if needing time to generate next response (PwPPA)	A +				18 (F)	0.33	**1**^a^
	To prompt / use open ended questions to encourage expanded responses (CP)	NA				4 (F) 14 (B)	6.3 0	**7**^a^ **0.3**^a^
8.01	To use non-verbal communication / gesture to indicate understanding (PwPPA)	A +	36.31	59.1	22.79	20 (F)	2.33	0.33
	To feel less frustrated in conversations (PwPPA and CP)	A						
	To have strategies for managing negotiations in conversation (PwPPA and CP)	A +						
6.01	To use more meaningful gesture in conversation (PwPPA)	A +	36.31	63.7^a^	27.39	20 (F) 22 (F)	12 0	**17.67**^a^ **0.33**^a^
	To use more writing and drawing in conversation (PwPPA)	A +				17 (F)	11.67	10.67
	To use more key words and automatic phrases in conversation (PwPPA)	A +				25 (F)	3	**3.33**^a^
6.03	To use intonation more to indicate agreement/disagreement, opinion, feelings and mood (PwPPA)	A +	38.2	49.02	10.82	26 (F)	0	**0.33**^a^
	To use eye gaze to point to support shared attention (PwPPA)	A				27 (F)	0.67	**3.33**^a^
	To use props/objects to support conversations (CP)	A				21 (F)	2.7	**6.7**^a^

*CP* communication partner, *PwPPA* Person with Primary Progressive Aphasia, *NA* not achieved, *A* achieved as expected, *A* + achieved more than expected, *A* +  + achieved much more than expected, ^a^more than 1 SD from the published *t*-score mean (Turner-Stokes, [41]), *F* facilitator behaviour that enhances the progressivity of conversation, *B* barrier behaviour that prevents progressivity, resulting in temporary breakdown of conversation

### Data to inform a sample size calculation

Having calculated an effect size of 0.86 for the AIQ-21 measure, at 80% power and using *α* 0.05, a sample size of 46 would be required. This was however a small randomised controlled pilot study and the effect size could therefore be inflated. Based on a more conservative effect size calculation of 0.5, at 80% power and using *α* 0.05, a sample size of 64 participants would be required.

### Safety

This was a low-risk behavioural intervention, and there were no adverse events or serious adverse events reported.

## Discussion

The primary aim of this study was to pilot the BCPPA program compared to a no speech and language therapy treatment control group over participating sites to establish for a future trial whether BCPPA can be delivered as intended in an NHS setting. This discussion will address findings in line with the specific objectives outlined in the introduction.

### Recruitment, eligibility and declining to participate (aims 1 and 2)

Time constraints on busy clinicians have been reported as a common barrier to recruiting participants [52]. Staffing issues, changes in service structure and COVID-19 restrictions plagued the study, and no further participants were recruited beyond the start of the pandemic. This situation is not dissimilar to that of other NHS-based trials of speech and language interventions, which report slower recruitment than anticipated in NHS-based RCTs [53, 54]. Ensuring the burden of research recruitment, consent and data collection does not impact on NHS research in the speech and language therapy profession is of vital importance to progressing treatment trials in this field.

The main reason that individuals with PPA were unable to participate in the study was failure to meet the inclusion criteria. This was due to prominent behavioural or memory disturbance associated with disease severity, implying people with PPA are referred to speech and language therapy when disease progression renders them less able to benefit from interventions. The two participants who withdrew following pre-intervention assessment also cited reasons associated with disease progression. Early referral to speech and language therapy for people with PPA can be hampered by misdiagnosis, for example the identification of psychiatric illness [55] or other dementia variants [4], and because other health professionals are not aware of the speech and language therapy interventions available throughout the PPA journey [25]. Offering speech and language therapy at the point of diagnosis would ensure people with PPA were connected with speech and language therapy services, who could consequently offer them support at timely periods in their disease journey.

A number of participants who did wish to participate were unable to identify an available CP who could attend face to face appointments. Trialling telehealth delivery methods such as video conferencing, which has been shown to be feasible in a case study of teleCPT by Beeke et al. [49], could improve accessibility for CPs who are unable to attend speech and language therapy sessions in person. TeleCPT could be combined with “out of hours” services for CPs, such as adult children, who may be working full time. Alternatively inviting people to participate in group therapy activities could provide access to new CPs for socially isolated individuals [56].

### Acceptability (aim 3)

Having been randomised to commence the intervention, no participants withdrew, indicating that both the process of randomisation and the intervention were acceptable. The intervention itself was generally viewed positively by participant dyads and local collaborators, as determined by self-report evaluation. Data collected from questionnaires highlighted that local collaborators felt some participants could have benefitted from more sessions, particularly those with more severe language impairment. This is important for implementation of the BCPPA intervention, suggesting dosage may need to be sensitive to disease stage.

It is notable that participant dyads’ ratings of sessions increased as the intervention progressed, despite the intervention not being what they had initially anticipated. In previous research, people with PPA and their families have reported that they do not know what speech and language therapists offer and have advocated for care pathways to support them in navigating their disease journey [57]. There is a general lack of awareness of the breadth of speech and language interventions amongst referrers [28] which may also contribute to mismatched expectations. There is an urgent need for care pathway guidance that can enhance knowledge of SLT amongst people with PPA and those in their support networks, including referrers.

### Treatment fidelity (aim 4)

The results of this study indicate it is feasible to adhere to the BCPPA intervention manual. The BCPPA manualised session plans, handouts and homebased tasks specify the standardised and tailored elements of the intervention [19, 20]. The high-fidelity rate confirms that the training sessions delivered were adequate for the local collaborators to adhere to the manuals provided. Methods to evaluate fidelity of this complex intervention were successful and should be considered for other speech and language therapy intervention studies [34].

### Primary outcome measure and sample size calculation (aims 5 and 6)

The aim of BCPPA is to reduce the impact of the person’s PPA on their lives, thus the AIQ-21 presents the most suitably sensitive outcome measure. Despite being designed for and with people with stroke aphasia, the AIQ-21 is a highly appropriate choice with its questions about communication, participation, well-being and emotional state. The AIQ results suggest that BCPPA has the potential to, just as it does for people with stroke aphasia, prevent the evolution of poor mental outcomes for people with PPA and their CPs [17]. The DEMQOL, the PPAs and the Zarit Burden Scale, common measure in dementia intervention studies, capture concepts related to quality of life, rather than change in communication behaviours (the focus of the intervention). The CCRSA, having been used in other PPA interventions studies, was chosen as a briefer alternative to the AIQ-21, with a focus on confidence over impact. Saldert et al. [58] highlight the challenge in CPT, of aligning the objective of the intervention with the projected outcome. They report that despite the main objective of CPT being more closely aligned with distal purposes of reducing the impact of a communication disorder (a distal outcome) in terms of how a participant feels, intervention effects are usually more likely to be demonstrated in proximal outcomes such as changes in interactional behaviours.

Changes in frequency of intervention-targeted interactional behaviours (a proximal outcome) can provide a measure of outcomes, when observed by an independent rater in a video recording. This method has been used by Best et al. [14] to demonstrate change in conversation behaviours following CPT for stroke aphasia. The current study also demonstrated a change in Interactional behaviours in the intended direction in more than half of participants. Importantly in the treatment fidelity literature, observing and recording the frequency of interactional behaviours is considered a component of monitoring a participant’s engagement with an intervention [34]. This is referred to as enactment, described as the process of putting plans into practice in daily life. Together with receipt, a participant’s ability to understand and perform the skills, these concepts describe how a participant has engaged in an intervention [33]. Investigation of enactment for the whole dataset in the pilot study is reported in Volkmer et al. [36].

Whether proximal or distal, there is little understanding of the most important outcomes from the perspective of people with PPA and their families. Certainly, goal attainment scaling, whereby a participant identifies personally important behaviours to change in therapy, demonstrated change in the intended direction in all except one of the goals set in this study. Recently, research has begun to explore the lived experience of people with PPA, their spouses and families [57, 59–61]. During PPI consultation as part of the current study, people with PPA, their families, SLTs and psychologists advocated that future research in PPA should explore the lived experience to understand what is important, and to inform the identification of a meaningful measure for use in intervention research.

### Implications for future research

Whilst this study has demonstrated that a coproduced CPT intervention delivered over four sessions within the NHS is acceptable and feasible for a future full trial, it has also raised further research questions.

As evidenced in this study, people with PPA are currently experiencing speech and language therapy access difficulties. Establishing a national care pathway would provide guidance to referrers on the role of the SLT and when speech and language therapy may be of benefit. Implementation of such a care pathway would require ongoing research, and its implementation would need review.

Earlier referral to speech and language therapy has been advocated by people with PPA and their families in the PPI work for this study [62]. Indeed, potential participants were often referred too late to participate in the BCPPA study. Early intervention has been identified as a factor in maintenance of therapeutic gains in other studies of PPA [63]. To support the development and implementation of care pathways for people with PPA, recruiting people as close as possible to the start of their disease journey will provide further information on optimal dosage and schedule of BCPPA.

It is imperative that the research community take account of the opinions of people with PPA and their CPs to understand meaningful outcomes of speech and language interventions for this group. As the research evidence in PPA, a relatively new area of practice, continues to develop, establishing a set of core outcome measures will be of benefit to maximise opportunities for comparison across studies. Incorporating the lived experiences and using consensus methods with expert researchers in the field to identify, agree and commit to using these measures will be a priority. This will consequently inform a future phase III full trial of BCPPA to establish whether the BCPPA intervention is effective for people with PPA.

Criteria to proceed to a full trial, set in advance of this randomised controlled pilot study, have been met, thus warranting a future full trial examining the effectiveness of BCPPA. In order to recruit an adequate sample size, it will be important to ensure adequate research resources, such as dedicated research staff who can recruit, consent and assess participants, to reduce the pressure on local collaborators. Considering the current UK prevalence estimates for PPA are approximately 2300 people, this randomised controlled pilot study has captured a potential pool of 2.6% and recruited closer to 1% of these. A future full trial would aim to recruit closer to 2.8%. Focusing on key national centres and diagnostic memory clinics would enable recruitment at a much earlier stage when people may be better able to participate. This will also allow for analysis of data according to severity of PPA variant based on neurological and speech and language evaluation.

## Limitations

Given the small and heterogeneous sample, these results must be interpreted with caution. The bespoke nature of BCPPA allows the intervention to be tailored to the individual needs of the dyad, whilst still delivering the core components of the intervention. Importantly, however, only one person with svPPA was recruited, likely due to the common occurrence of behavioural difficulties in this variant, which may prohibit inclusion [3]. In fact, many potential participants did not fulfil the inclusion criteria for the study as they were too progressed in their disease journey to participate in the intervention. This is likely attributable to difficulties that people with PPA in the UK have in accessing speech and language therapy in a timely manner [28]. This resulted in a smaller sample size than anticipated. This, in turn, resulted in significant heterogeneity across participants in terms of language profile and communication difficulties. This makes it difficult to compare participants to one another, thus a range of outcome measures were piloted across language, communication and quality of life to identify the most sensitive measure across participants. The large number of measures may have contributed to anxiety experienced by two participants who chose to withdraw following pre-intervention assessment. This emphasises the importance of selecting fewer, suitably sensitive, core outcome measures for a future full trial.

Other study limitations include the inability to mask participants to group allocation, a common barrier in behavioural studies. Similarly, it was not possible to mask local collaborators delivering the intervention. For this reason, post-intervention assessment was completed by pairs of junior researchers (student SLTs) masked to group allocation. Constructing an active control arm in a future trial will both enhance scientific analysis and assist in masking as well as addressing the ethical implications of a no treatment arm. Despite the intervention being deliverable within four sessions, many participating local collaborators were unable to offer these on a weekly basis. Systematic data on the intensity of intervention delivery were not collected. As this is relevant to dosage, it would be beneficial to consider in a future trial [17].

Multiple post-intervention reassessment points would provide information on maintenance of treatment effects following intervention. It is ambitious to expect a 4-week intervention to result in immediate gain, and there is some evidence from the chronic disease literature that treatment effects following self- management interventions may be more observable over a longer-term period following an intervention, as participants establish proficiency in using in daily life what they have learnt [64].

## Conclusions

The first randomised controlled UK pilot study of a CPT program for people with PPA and their families demonstrates BCPPA is a promising intervention. Two thirds of participants over-achieved goals, the intervention was acceptable to participants and SLT collaborators and treatment fidelity was high. Though fulfilling continuation criteria, this research has identified unanswered questions that should be addressed prior to proceeding to a full trial.

## Supplementary Information

**Additional file 1.** CONSORT 2010 checklist of information to include when reporting a randomised trial*.

## Data Availability

Video recorded datasets generated and/or analysed during the current study are not publicly available due to ethical restrictions related to sharing of video data. Other datasets are available from the corresponding author on reasonable request and subject to consents.

## Abbreviations

BCPPA: Better Conversations with Primary Progressive Aphasia
CP: Communication partner
CPT: Communication partner training
ISRCTN: Numerical identification of randomised controlled trials
MRC: Medical Research Council
NHS: National Health Service
PPA: Primary progressive aphasia
RCSLT: Royal College of Speech and Language Therapists
SLT: Speech and language therapist
UCL: University College London
